# Letter to the Editor Concerning the Publication: “Meta-Analysis of Enhanced Recovery Protocols in Bariatric Surgery”

**DOI:** 10.1007/s11605-018-3828-4

**Published:** 2018-05-31

**Authors:** Piotr Małczak, Magdalena Pisarska, Michał Wysocki, Piotr Major, Michał Pędziwiatr

**Affiliations:** 1 2nd Department of General Surgery, Jagiellonian University Medical College, Kopernika 21, 31-501 Kraków, Poland; 2 Centre for Research, Training and Innovation in Surgery (CERTAIN Surgery), Krakow, Poland

Dear Sir,

We have read with interest the study by Ahmed et al. entitled “Meta-Analysis of Enhanced Recovery Protocols in Bariatric Surgery”.^1^ We would like to congratulate the authors, Ahmed et al. for a well-written paper involving 13 studies in their meta-analysis. While in general the methodology of the study tries to follow current guidelines for performing systematic reviews, we get the impression that some important steps are either not clear or incorrectly performed. Due to significant differences in the main results of this review and previous meta-analyses, we have some questions to the authors.

First of all, why none of the diagrams provides data for the analysis? In our opinion, this practically deprives readers of the opportunity to analyze the results and the review is no longer transparent. It is especially important in cases when studies do not provide data on overall morbidity. We do not know what numbers were in the analysis or were the authors contacted to assist with this lacking data. Simple adding up intraoperative to postoperative complications or surgical to non-surgical complications in some of included studies may lead to overestimation corrupting the final results.^4^–^6^ If accurate data is unavailable, such study should be excluded from data synthesis to avoid this situation.

Secondly, why in the methodology, the authors mention the threshold for using fixed-effect model of meta-analysis is *I*^2^ < 50% and change the assumption when showing the results? Figure 3b, which presents data for morbidity, shows *I*^2^ of 54% and fixed-effect model being used or Fig. 3c, presenting data for operative time with *I*^2^ of 96%. This has introduced serious bias because the meta-analysis of included studies using random-effects model may have shown different results. This is very serious flaw in this meta-analysis which dramatically changes the results. Previously published systematic reviews including the same studies showed contradictory results.^2^,^3^

And finally, why the results of the study by Barreca et al. are not included in the meta-analysis? Although it appears in the diagram, it is not taken into further calculations because the data for this study was not introduced.

In our understanding any systematic review must be performed without any flaws and be as transparent as possible. Nowadays, we all rely our clinical decisions on meta-analyses and well-designed trials. This issues question the reliability of the results and for this reason, we are forced to ask for a strong reaction either from the Editors or Authors. As surgeons and ERAS enthusiasts, we strongly believe that multimodal perioperative care is beneficial for bariatric patients. However, as methodologists who analyze the available evidence, we must agree that it is sparse and does not fully show what we see or would like to see in our daily clinical practice. We do not believe further trials are needed to fully confirm benefits of ERAS because randomization to control group would expose patients to rather outdated practices. We must accept what has been published but at the same time, we must act according to methodological guidelines.
